# A Pipeline for Phasing and Genotype Imputation on Mixed Human Data (Parents-Offspring Trios and Unrelated Subjects) by Reviewing Current Methods and Software

**DOI:** 10.3390/life12122030

**Published:** 2022-12-05

**Authors:** Giulia Nicole Baldrighi, Andrea Nova, Luisa Bernardinelli, Teresa Fazia

**Affiliations:** Department of Brain and Behavioral Sciences, University of Pavia, 27100 Pavia, Italy

**Keywords:** imputation pipeline, mixed data, unrelated subjects, trios, LD-based method, SNPs

## Abstract

Genotype imputation has become an essential prerequisite when performing association analysis. It is a computational technique that allows us to infer genetic markers that have not been directly genotyped, thereby increasing statistical power in subsequent association studies, which consequently has a crucial impact on the identification of causal variants. Many features need to be considered when choosing the proper algorithm for imputation, including the target sample on which it is performed, i.e., related individuals, unrelated individuals, or both. Problems could arise when dealing with a target sample made up of mixed data, composed of both related and unrelated individuals, especially since the scientific literature on this topic is not sufficiently clear. To shed light on this issue, we examined existing algorithms and software for performing phasing and imputation on mixed human data from SNP arrays, specifically when related subjects belong to trios. By discussing the advantages and limitations of the current algorithms, we identified LD-based methods as being the most suitable for reconstruction of haplotypes in this specific context, and we proposed a feasible pipeline that can be used for imputing genotypes in both phased and unphased human data.

## 1. Introduction

Genotype imputation is a computational and economical technique that enables the genotype of genetic variants which, for example, have been discarded during the quality control (QC) steps to be retrieved, and ex novo non genotyped DNA stretches to be inferred [1]. Single nucleotide polymorphism (SNP) genotyping arrays can contain up to 2.5 million markers, which covers only a small fraction of the complete human genome [2], and 90% of known SNPs are highly correlated with at least one typed variant [3]. In this context, imputation represents a cost-effective strategy for gaining high-density genotypes. It is a powerful tool that can be used to increase statistical genomic coverage [4], therefore facilitating the meta-analysis of studies which make use of genotyping obtained with different panels (chips) [5]. By dramatically increasing the number of genetic markers that can be tested for association, imputation has a crucial impact on the identification of disease-associated genetic regions and causal variants [6,7]. In the most recent era of high-resolution genome-wide association studies (GWASs) [1], which is a key step of genotype imputation [7], it is now standard practice to increase genome coverage and improve accuracy of genomic selection of SNP array data [8], thereby enabling high resolution fine-mapping of candidate genomic regions [6]. Thus, it is important to clearly assess which computational approach for imputation is most suitable for a given type of target data. 

Genotype imputation uses the concept of a haplotype, which is the combination of alleles on a single chromosome that is inherited together from a single parent [9]. Although haplotypes are not directly observed through genotyping, they can be inferred and reconstructed by phasing. Phasing is the process of statistical estimation of haplotypes; it involves using the genotyped (observed) data and their corresponding probabilities to establish whether a particular allele resides on one or the other of the two paternal chromosomes. In the absence of the parents’ genotypes, phasing relies on a statistical procedure. Other than for imputation [4], estimated haplotypes can be used for inferring ancestry [10], demographic history [11], or detecting causal variants [12]. Imputation methods work by comparing the estimated haplotypes of the study sample with denser reference haplotypes. By matching haplotypes between the two panels, the genotypes of unobserved variants in the study sample can be obtained. Since matches are not unique and the imputation methods average over many possibilities, this results in the production of a probability distribution for the unobserved alleles/genotypes [13]. 

Imputation can be implemented on both raw unphased genotyping data [14] and reconstructed phased haplotypes [15]. The main difference between these data types is the information that phased data carries within the analysis, i.e., the possibility to analyze compound heterozygotes, to measure allele-specific expression, and to identify variant linkage. Phasing data leads to a better imputation accuracy, which is estimated by calculating the similarity rate between in silico imputed variants and true genotypes employing cross-validation methods [16]. Furthermore, using phasing data, the computational time of imputation is significantly reduced. In addition, compared to unphased data, prephasing haplotypes represents a better strategy for imputing variants that were not genotyped during the experiment, hence producing a lower percentage of missing data after imputation [1,17,18]. Therefore, phasing is an important aspect to consider when writing an imputation pipeline, since it can enlarge the number of typed variants. 

An imputation software employing reconstructed haplotypes requires the pre-imputation computational processing step of phasing to be performed [19]. Haplotypes can be reconstructed for both related and unrelated individuals. For unrelated individuals, the study sample size represents a critical factor for a successful phasing, because in the absence of family members, similar combinations of SNPs to be phased are not always likely to be encountered [20]. The suggested overall sample size for unrelated individuals is over 50; the bigger the sample size is, the longer the haplotypes that can be reconstructed [21]. The recommended strategy for unrelated individuals is to perform phasing with haplotype frequency information from a reference population which has been more densely genotyped or sequenced [19,22,23], such as from the HapMap Project and the 1000 Genomes Project [24,25,26]. For related individuals, estimation is performed by considering both the haplotypes that are shared between family members and the haplotype frequency information of the reference population. The reference panel from the 1000 Genomes Project has more than 80 million variants in 504 individuals from 26 populations and is one of the most used panels due to its large sample size, population diversity, and free access. The Haplotype Reference Consortium (HRC) contains more individuals (N = 32,488), mainly with European ancestry, and is ideal for imputation of low-frequency and rare variants in European samples [27].

The proper selection of the reference panel represents a very crucial practical aspect that influences imputation performance, both in terms of the size of the reference panel and the ethnicity of the represented population. In fact, a larger reference population provides more reference haplotypes so that the target variants can be more easily matched to them, thereby increasing the reliability of the imputation [28]. 

Despite many large-scale whole-genome sequencing (WGS) projects that have been developed in the last few years, haplotype reference panels are not available for most of the world’s population, and since haplotype imputation requires ethnicity-matched references, new software were developed to overcome this issue. For example, HiFi, a cost-effective software, uses existing unphased genotype datasets as references to generate a statistical haplotype reference panel [29].

Furthermore, depending on the reference panel used, the imputation of low and rare variants ca also be performed [30]. Most algorithms and software perform well when dealing with common variants but not when inferring low-frequency and rare variants [31]. The latter plays a key role in human diseases and can capture the proportion of unexplained genetic components of complex traits, the so-called ‘missing heritability’ [32]. Although imputation increases the number of these low-frequency and rare variants, its accuracy is usually low [33,34,35] and mainly depends on both the software and the reference panel used. This latter issue can be solved by increasing the sample size and by using population-specific haplotype reference panels [24] that are well-matched in terms of ancestry. Since the number of population-specific differences increases as allele frequencies decrease [36], these strategies can be used to improve the genotype imputation, especially for low-frequency and rare variants [36]. 

Therefore, researchers willing to focus on rare variants may often use next generation sequencing (NGS) data, which is typically more expensive than SNP chip genotyping, to obtain WGS SNP markers. Despite the economical advantage of using SNP chips compared to sequencing data, SNP chips cannot capture all the relevant genomic information, particularly if the variants on the chip array are not in LD with the causal mutations. 

One proposed option that offers advantages over SNP arrays is to impute from many low-coverage whole-genome sequencing (LCWGS) individuals [37]. In fact, LCWGS, in a cost-efficient manner, allows for better genotyping of low-frequency variants without losing power at common variants. Furthermore, LCWGS is optimal for populations not specifically targeted by commercially available SNP array platforms. Imputation works by refining the genotype likelihoods of low-coverage sequencing and filling in the gaps between sparsely-mapped reads using a reference panel of haplotypes [37]. 

A new method and open-source software called the genotype likelihoods imputation and phasing method (GLIMPSE) has shown a remarkable performance using LCWGS data for both European and African-American populations [37]. It drastically reduces the computational cost via a new powerful linear time sampling algorithm which causes the computational imputation time to decrease as the size of the reference panel increases. It allows accurate imputation using large reference panels, which is an important aspect to consider given that larger panels are constantly being made available. Imputation accuracy using GLIMPSE is the result of not only the size of the reference panel, but also the sequencing coverage and the ancestry of the reference panel in relation to that of the target samples. 

However, additional methods other than GLIMPSE are needed and should be developed to improve genotype likelihood calculations, running times, and data management. Further improvements may involve the extension of the imputation by leveraging information from the target dataset in cases when there is no reference panel. This may be especially relevant when the reference panel is considerably smaller than the study sample as it means that the study sample can serve as their own reference panel [37]. Many software programs, such as FImpute [38], which is suitable for dense sequencing data [28], and another new approach called RefRGim [39], which uses convolutional neural networks (CNNs) to reconstruct a reference panel genetically similar to the study individuals, were also found. It compares the sequence similarity between the study sample and original reference panels and provides the reference haplotypes with the best similarities, thus achieving high accuracies, particularly for low-frequency and rare variants.

The imputation accuracy of both common, low-frequency, and rare variants can be further improved by using population-specific panels that allow for the identification of variants that would not have been found otherwise [33,40,41,42], like what happened in the UK10K project, where the British population-specific reference panel combined with the 1000G Project panel facilitated the discovery of many phenotype-associated genetic variants [43].

Ideally, the chosen imputation algorithm should be fast and efficient [17], should allow access to a high-performance cluster and to a reference panel of sequenced genomes [44] and, most importantly, should be as accurate as possible [45]. Tools and algorithms that help to solve the genotyping imputation problems are increasing in number [20,21], in accuracy performance [46], and in their ability to conduct multi-level complexity imputations [11,23,24]. Software descriptions are widening in scope and include exhaustive definitions of their features and the type of data on which the methods can be applied [21]. 

If imputation is performed on phased data, it is necessary to define a method for phasing haplotypes according to haplotypes’ frequencies, structures of haplotypes and relatedness.

Broadly, software can be mainly categorized into two groups on the basis of the method used to reconstruct the haplotype: identity-by-descent (IBD)-based and linkage disequilibrium (LD)-based methods [47]. Within related individuals, the presence of relationship information and a low rate of recombination among offspring means that information on haplotype patterns is easier to reconstruct. For unrelated data where we do not have information on either the relationship between individuals or the recombination rate, the approach is to reconstruct recombination patterns that have occurred by comparing genotypes with only a reference panel, a method with a higher rate of error than in related individuals. Then, the appropriate imputation method with phased data estimates the genotype for each subject based on the most probable haplotype. 

On the other hand, with unphased data, the software for imputation calculates the probability of the haplotype being present in the population from which it is sampled, and then estimates the missing genotypes frequencies, using both allelic frequencies and comparisons to the reference panel. Having mixed data (trios/unrelated) results in wider margins of error than when using unphased data. Furthermore, it can take a longer time for imputation. Thus, pre-phasing is recommended if possible. 

Both IBD- and LD-based methods are suitable in the context of mixed data [24,44]. Specifically, IBD-based methods use shared genomic stretches between individuals to directly phase each individual’s genotype [25]. Since it performs well on distantly related subjects [25] and does not always require a preliminary haplotype phasing step, it is able to work on unphased genotypes. Observed genotypes in each sample are compared with haplotype information of a reference sample, which should always be provided. Chromosome regions shared between the study sample and the individuals from the reference panel are identified, and finally, haplotypes sharing information are combined to fill the unobserved/missing genotypes in the study sample. On the other hand, LD-based methods [48] mostly use pre-phased input data on which a pre-imputation step that involves haplotype reconstruction has been performed. LD-based methods can be applied using different model settings. One possible approach employs Markov-Chain Monte Carlo (MCMC) [49,50] resampling methods, e.g., Gibbs sampling [51], based on the Metropolis Hastings algorithm [52,53]. In this approach, the unobserved genotypes are considered as random variables and their conditional distribution is evaluated using the observed genotypes and the reconstructed haplotypes. 

When planning a data analysis pipeline for imputation, it is firstly important to clearly consider the type of data, i.e., related individuals (i.e., trios, duos, extended pedigree) or unrelated individuals (i.e., case-control) or both (i.e., mixed data) that will be used so that the appropriate software required to set up the most suitable pipeline can be chosen. Furthermore, other criteria which classify the different imputation software should be considered. These criteria include: (i) computational efficiency [54]; (ii) the imputation algorithm used; and (iii) the achieved accuracy [45,53].

Although the literature on imputation strategies and their applications is exhaustive, it lacks a clearly established pipeline to implement phasing and imputation for SNP arrays when dealing with a targeted sample that consists of mixed type data (i.e., related and unrelated individuals) that is aimed at reducing imputation bias and its propagation.

In this research, we reviewed the existing literature regarding possible approaches and available software for phasing and genotype imputation in mixed-sample settings, particularly when related individuals belong to trios (both parents and their offspring) or even duos (just one parent and their offspring). Here, we did not concentrate on extended families in which there are many familial relationships, including, for example, grandparents and other relatives, as the relevant tools have been deeply investigated in the literature [25,55]. Our aim was to identify an easy and feasible pipeline that could be applied in the specific context of targeted samples containing mixed data and reduce bias propagation deriving from mis-specified imputation tools.

## 2. Material and Methods 

We reviewed the existing literature on genotype imputation for mixed type human data by adopting the structure found in Grant and Boot’s guidelines [54] and the macrostructure of the PRISMA [56] guidelines for systematic reviews and meta-analyses. We then defined an affordable genotype imputation pipeline that can be adapted for use with a targeted mixed sample, thereby reducing possible propagation bias for the following analyses. 

The steps followed for the critical review of the existing literature are summarized below:

- *Definition of the aim and research question*
  Our main research question was: “*To define our feasible pipeline, which is the algorithm, and consequently the software, that can be used for performing genotype imputation and, if required, pre-phasing in the case of mixed type data?*”.

- *Definition of the searching strategy and keywords*
  The search strategy was based on the inclusion of papers which contained the following topics: (i) imputation and phasing methods for genotypic data in trio or nuclear families and/or (ii) imputation and phasing methods in unrelated subjects; papers related to both (i) and (ii) had to contain (iii) free software for imputation and/or phasing from a SNP genotyping platform. The keywords considered were: genotype imputation, haplotype phasing, haplotype estimation, freeware, Hidden Markov Model (HMM), trio, case-control, LD-based method, IBD-based method, nuclear data, family data, related subjects, unrelated subjects, SNP genotyping platform, and SNP array.

- *Definition of information sources*
  To retrieve the documents, we used NCBI-PubMed and Google Scholar, selecting both original research articles and reviews. Our literature search was carried out in February 2022.

- *Selection process: definition of the inclusion criteria*
  The inclusion criteria for the selected papers were the following: (i) documents published in English; (ii) documents that were open access; (iii) documents that clearly described the methods and algorithms used or discussed; and (iv) scientific papers, software manuals, and online tutorials, which refer to published scientific works in which a freeware is used. No limits were set as to year of publication. Two authors (GNB, TF) independently screened the full text for all the papers, as the abstracts did not allow us to obtain the information required.

- *Extraction of qualitative data*
  All the information gathered was reported in a table containing: (i) the article reference; (ii) the type of study; (iii) the topic covered, i.e., phasing, imputation, or both; (iv) the type of data analyzed; d) the software used; and (v) the algorithm/s on which the software relies/y.

- *Reporting synthesis: summary of qualitative data*
  The qualitative synthesis was carried out by considering and discussing software features and algorithms most suitable for mixed data (trios, duos, and unrelated subjects) following the PRISMA guidelines [56] and critical review description outlined by Grant and Boot [54]. Our aim was to summarize how to implement software for haplotype phasing and genomic imputation while controlling for bias introduced by imputation on mixed data.

## 3. Results

### 3.1. Critical Review

**Figure 1:**
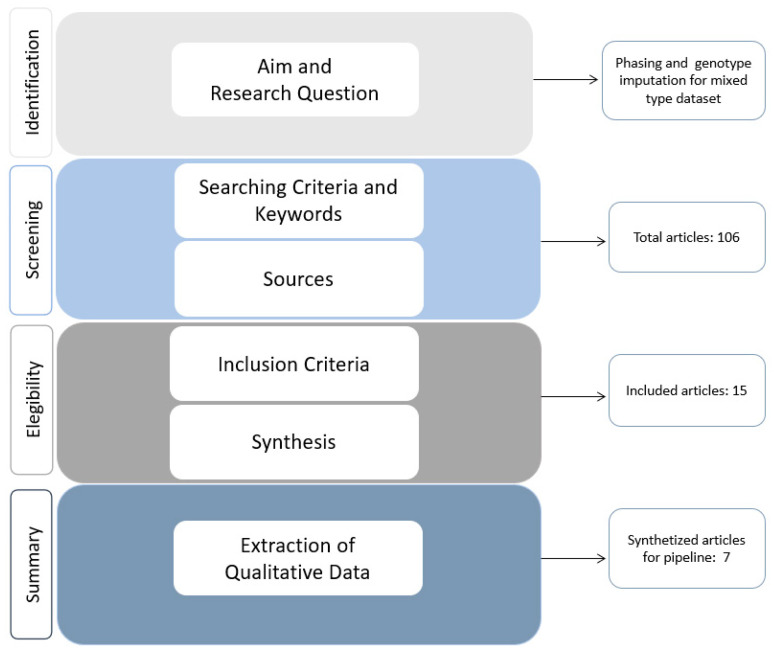
Workflow followed for the critical review. The critical review steps were grouped into four areas: identification of the problem, screening of related literature, eligibility of scientific works, and summary of the qualitative data according to the criteria declared in the Material and Methods section.

To answer our research question which was: “*To define our feasible pipeline, which is the algorithm, and consequently the software, that can be used for performing genotype imputation and, if required, pre-phasing in the case of mixed type data?*”, we searched for all the relevant scientific papers in the literature following the criteria described in the Material and Methods section. In Figure 1, the workflow of this study is graphically represented, and the four macro sections (identification, screening, eligibility, and summary) are summarized also following the steps described in the Material and Methods section. For each macro section, which are represented by the left white transversal labels, the steps were reported in the coloured central areas and their respective partial results were listed in the right white boxes. As reported in Figure 1, we initially found 106 scientific papers; after carefully reading and screening each of them, we found that only 15 papers fulfilled the chosen inclusion criteria and were selected for the critical review synthesis. Only seven out of the fifteen scientific works were useful for the qualitative synthesis as they reported a detailed description of tools and/or software that effectively answered our research question. 

In Table 1, the list of the initially screened 15 papers, in chronological order of publication, was reported. The type of analysis performed (phasing, imputation, or both), the type of data used for the analysis, and the software and algorithm implemented were also indicated. The 15 papers and manuals included: (i) nine scientific papers describing software and algorithms implemented [15,55,57,58,59,60,61,62,63]; (ii) two software documentations [52,64] that exhaustively described software functioning and which data type can be implemented; (iii) two scientific papers highlighting the different characteristics for phasing algorithms [25,65]; and (iv) two critical reviews on the quality assessment of imputation methods [17,24]. Seven out of the fifteen papers were finally selected (indicated in bold in Table 1) as they contained an exhaustive description of all the information required for application to a mixed type dataset (see Material and Methods).

Specifically, the work from Stephens et al., 2001 [65] compared different algorithms for phasing that allow researchers to reconstruct genome information using LD patterns in the study sample and reference populations [66,67] and are suitable for both related and unrelated individuals [68,69]. As explained in [65], the methods used for haplotype estimation range from the expectation maximization (EM) algorithm [68] to LD-based methods (e.g., HMM). Briefly, considering G to be the observed individual’s genotype, the EM algorithm [70] finds the set of unknown population haplotype frequencies (F) that maximize the likelihood function $L\left(F\right)$, which is defined as the probability of observing the individual genotypes given the population haplotype frequencies and under the assumption of Hardy Weinberg Equilibrium (HWE):

(1) $$L\left(F\right)=Pr(G|F)=\Pi_{i=1}^{n}Pr(Gi|F)$$

where *Pr(Gi|F)* is calculated as the sum of all the possible haplotype frequencies from the set of all (ordered) haplotype pairs, consistent with a multilocus genotype. A limitation of using the EM algorithm is that although it is suitable for handling small numbers of loci, it becomes computationally expensive and loses accuracy when used for larger numbers of markers. Therefore, LD-based methods represent more accurate and widely used methods for haplotype estimation. They are well suited to describe the evolution of observable events [71] that depend on latent factors which are not directly observable in the context of sequences of Markovian Chains. The observable events are represented by the sequenced genotypes (symbols), while the invisible factors underlying the observable events are represented by the haplotypes (states). An HMM consists of two stochastic processes, namely, an invisible process of hidden states and a visible process of observable symbols. The hidden states form a Markov chain [72] and the probability distribution of the observed symbol depend on the underlying state. For this reason, an HMM is also called a doubly embedded stochastic process [73], which refers to its modeling of observations in these two layers, one visible (sequenced genotypes) and the other invisible (haplotypes). HMMs have been shown to be very effective in representing biological sequences (e.g., nucleotides on the genome, amino acids in proteins) [73,74]. Implementation of HMMs employs resampling algorithms such as Viterbi [68,69] or Gibbs sampling [15,55,58,60,75]. Following a Gibbs sampling approach, everyone’s haplotypes are updated based on the current estimates of haplotypes from all other samples and are subsequently used for the conditional distributions of the Gibbs sampler. Here, for each individual *i*, any haplotype’s probability *Pr*($H_{i})$, consistent with the genotype $G_{i}$, can be calculated according to Markovian Chains as:

(2) $$Pr(H_{i}\;\;|G,\;H_{i-1})\;\;\;$$

A Gibbs sampling method is then used to construct a more complex algorithm that involves the presence of multilocus genes in a sample of observed genotypes [73]. Compared to the EM algorithm, HMM can therefore be applied to very large numbers of loci and can naturally capture the uncertainty associated with haplotype reconstructions.

Thus, we concluded that in our context the most suitable method is LD-based and employs the HMM setting for reconstructing haplotypes given its previously described advantages over the EM algorithm [15,55]. Imputation methods are based upon the HMM and have computational constraints due to an intensive sampling process. The deterministic approach underlying HMM makes use of both family and population information [38]. If individuals are related and, therefore, share haplotypes that may differ in length and frequency based on their relationships, this approach leads to the consideration of pedigree information and exploitation of close relationships by searching for long haplotype matches in the reference group using overlapping sliding windows. The search continues as the window size is decreased in each chromosome sweep, which allows more distant relationships to be captured. As for unrelated individuals, genotypes are imputed by recovering [76] the genotypes of untyped loci using information from reference individuals that were genotyped with a higher density panel, a process which is computationally challenging.

For this reason, for this qualitative synthesis, we excluded the paper by Abney and Elsherbiny (2019) [57] that describes Kinpute software, an IBD-based method [77], since important issues may arise when imputing based on IBD shared regions [78,79]. In fact, when having only trios as family data, implying very close relationships, the effect of shared regions could be overestimated [17,80]. Specifically, the farther the relationships between individuals are, the smaller the size of the shared branches of chromosomes that are likely to be encountered [77], especially when compared to closer relationships [79,81]. For the same reason, we also discarded from our review the works by Kong et al., 2008 [25], since it described a long-range IBD-based method, and by Yun et al., 2008 [17], since it contained a review of IBD-based imputation methods. The AlphaImpute package proposed by Hickey et al. [63] remains an interesting proposal for dealing with related individuals with pedigree structure. It can be used as a tool for long-range phasing, therefore helping in the context of extended families. Like for Kinpute software, it performs well when individuals share shorter percentages of DNA [57], because information is reconstructed using an IBD-based, not an LD-based, approach [77]. For these reasons, it is not properly applicable in the context of small parent–offspring trios and duos. Alternatively, it can also perform imputation with the HMM method, which is particularly useful when phasing information is not available or when imputation is required in unrelated populations [1].

We also decided not to consider the paper by Money et al., 2015 [59] in which an LD k-Nearest Neighborhood (LD-kNN) method [82,83], as implemented in LinkImpute [59], was described. The basic idea of the kNN setting is to compute a distance measure between each pair of observed markers (e.g., Euclidean) and the number of contributing neighbors for each prediction, i.e., the k hyperparameter of the kNN algorithm, based on the non-missing variables. The k-nearest observations that have non-missing values for that variable are used to impute a missing value through a weighted mean of the neighboring values [84]. LD-kNN shares some similarities with IBD-based method settings for the issues concerning the analysis of trio data [85]; therefore, this method is better suitable for extended families or unrelated individuals than trios. We further excluded from our review the works by Delaneau et al., 2013 and Khankhanian et al., 2015 as they [60,62] deal with software, i.e., MACH and Impute2, that perform well when imputing genotypes in unrelated subjects but do not perform well with familial structures. 

Lastly, we excluded the work by Scheet and Stephens (2008) [64] as we decided to include in the proposed pipeline Beagle software, as described in Browning et al., 2007 and Browning et al., 2021 [52,61], instead of fastPHASE software which is mentioned in [46]. Nevertheless, fastPHASE can be a good alternative [86] for mixed data genotype imputation, as described in a comprehensive assessment of quality provided by Shi et al., 2019 [24], as well as in [87] (this latter study was not included in this critical review synthesis since the authors only presented a description for extended relationships, not for trio data samples). Although Beagle and fastPHASE include similar features [88], such as employing the HMM approach for haplotype estimation [89], some slight differences exist between the two methods. For example, fastPHASE relies on a fixed number of haplotype clusters to form underlying hidden states in the Markov Chain, while Beagle allows the haplotype clusters to dynamically change to better fit localized LD patterns [80]. In addition, Beagle’s memory requirements can be controlled by adjusting the length of the sliding marker window. Given the above considerations, we focused on Beagle as an LD-based imputation software for our proposed pipeline. 

**Figure 2:**
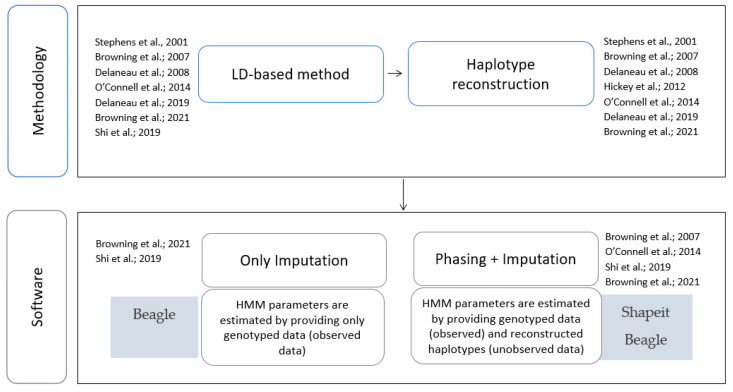
Qualitative synthesis. In this figure, the chosen 7 [15,25,52,55,58,61,65] out of 15 scientific works on which we established the qualitative synthesis for our pipeline are represented, along with highlights of their utility in the choice of pipeline steps.

Figure 2 reported the summary of the qualitative synthesis of the seven selected papers in light of our research question and proposed pipeline. The figure was split into: (i) an upper box labelled “Methodology”, which listed the papers referencing LD-based methods and, among these, those which described an HMM approach for haplotype reconstruction; and (ii) a bottom box labelled “Software”, which listed papers referencing software to be used for imputation, i.e., Beagle, and for phasing plus imputation, i.e., Shapeit plus Beagle.

### 3.2. Pipeline for Genotype Imputation

**Figure 3:**
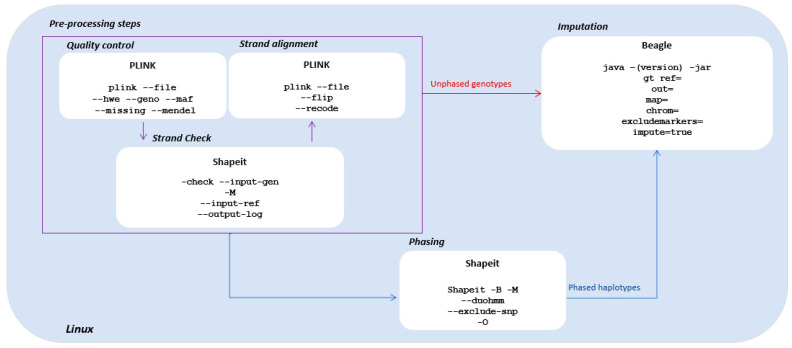
Pipelines for genotype imputation on mixed type data. The key steps of the pipeline are summarized in this scheme: pre-processing steps (top left region enclosed in the purpose square), phasing and imputation (blue arrow) for phased data or, alternatively, direct imputation without phasing (red arrow) for unphased data. Only essential commands and options are reported. A detailed list of all the functions and options is available on the respective software websites.

Here we reported our proposed pipeline that can be used for genotype imputation on both unphased and pre-phased mixed data. In Figure 3, the steps involved in the pipeline, the software used, and the respective code are schematically reported.

#### 3.2.1. Pre-Processing Steps

To ensure correct data imputation, it is important to perform some routine QC checks (pre-filtration): (i) testing HWE to check [90] whether allele and genotype frequencies in a population are constant among generations, assuming the population that generated the sample is not under evolutionary influences [91], and that deviation from HWE indicates genotyping artifacts; (ii) calculating minor allele frequency (MAF) [92], which is the frequency at which the minor allele occurs in the population [93]; (iii) calculating the percentage of missing data, which is essential to investigate the distribution of call rates by the marker and the sample and the overlap between the two; and finally, (iv) verifying the presence of Mendelian errors. This last check is performed only on related individuals [94] to identify any Mendelian inconsistency between genotyping information and pedigree structure. All the mentioned QC steps could be performed using PLINK software [95]. 

Another important step involves checking the strand alignment to verify the presence of ambiguity over which strand to look at among observed SNPs. Since DNA is composed of two antiparallel strands, it is fundamental that the study sample dataset is aligned with the population reference panel of haplotypes used. Strand check could be performed by using Shapeit software [60,96], and in the case of misaligned sites between panels (observed vs. reference), the allele flipping, if possible, could be performed by using PLINK. 

The threshold should be carefully set as a QC that is too stringent can remove too many variants. Therefore, to avoid variant exclusion, less stringent QC may be required depending on the study aim and sample. Commonly used cut-offs for the SNP filtering criteria are MAF > 1–5%, HWE *p*-value > 10 ^− 6^–10 ^− 4^, and call rate > 90–99% [97,98].

In the top-left purple box of Figure 3, the pre-processing steps of data quality control (QC), strand check, the subsequent strand alignment, and the corresponding software and software code used were reported. Specifically, for the QC steps, PLINK functions *--hwe*, *--maf, --geno*, and *--missing* are used to test HWE; to calculate the MAF, the threshold of which is identified between 1–5% for rare variants; to calculate the missing rate per genotype and individual; and to identify markers and/or individuals with excessive missingness rates, respectively. Excessive missingness happens when there are high percentages of missing data (higher than 15–20% among individuals in the whole dataset), which is the typical scenario where imputation is not recommended. In general, in random settings, statisticians are encouraged to perform imputation when the rate of missing is 5–10%. In addition, it is a rate at which algorithms for genotype imputation report high discordance. It happens mostly in the case of unrelated subjects, because for related ones, higher rates of missing data can be better handled. Furthermore, SNPs with call rates > 90%, which are also considered high percentages of missing data, can be removed. As previously mentioned, when analyzing related individuals only or mixed data, it is important to check for Mendelian errors (*--mendel*) and to eventually correct for them. Other QC steps, e.g., loss of heterozygosity [99], could also be performed depending on the study aims. The output file name can be customized by adding the *--out* option; while the *--file* option defines the input file to be analyzed. As for the strand check, the Shapeit function *--check* command is used. This command runs on different genotype input files, e.g., [100] Oxford format, that is specified using the option *--input-gen*. The *--map* and -*input-ref* options for specifying, respectively, the genetic map and the reference genome on which the check must be performed are also required. If the study sample and reference genome are built under different releases, the study sample must undergo a previous step using tools, e.g., UCSC liftOver (http://genome.ucsc.edu/cgi-bin/hgLiftOver, accessed on 15 March 2022) and CrossMap [101], to uniform the release. After the strand check, if misaligned sites between panels (i.e., study sample and reference genome) are identified, these sites need to be aligned before proceeding with imputation. If possible, misaligned alleles need to be flipped (strand alignment) in the study sample by using the PLINK function –*flip* and the option –*recode* for specifying the desired output file (for example genotype in Oxford or binary format) and the -*-out* option for customizing the output file name. Depending on the targeted data set, even moderate filtering can have a huge effect on imputation quality. Little or no SNP filtering prior to imputation appears to be the best strategy for imputing small to moderately sized datasets [30,98].

After the above reported pre-processing steps, the data are ready for imputation. Two scenarios can then be followed: (i) imputation on phased haplotypes or (ii) imputation on unphased genotypes. The difference between the two pipelines is that the LD-based method to reconstruct genotypes can either be processed before imputation by reconstructing haplotypes (with the pre-phasing step) or directly on genotypes during the imputation step (without the pre-phasing step). Both of the two applications are described in Section 3.2.2 and Section 3.2.3, below. 

#### 3.2.2. Imputation with Pre-Phased Haplotypes

To perform phasing, we identified the freeware Shapeit implemented for the Linux environment. An input genotype file, e.g., binary format (with options *-B*), and a genetic map of the specific chromosome (with options *-M),* are provided to perform phasing. Shapeit can phase genotype data from both related and unrelated individuals. The mixed sample can be phased together by adding the *--duohmm* option to correctly read pedigree information [102]. An HMM algorithm is employed by Shapeit to reconstruct unobserved genotypes [20]. This LD-based reconstruction process uses observed information (provided by observed genotypes) combined with the haplotypes of the reference data [96] to estimate HMM parameters and subsequently infer population haplotypes. It also estimates the probability of recombination events according to probabilities defined by the HMM [103]. When analyzing related individuals, the estimated HMM parameters are also calculated based on pedigree information. Other offset parameters, e.g., window size and allele frequencies, can be set for reconstructing haplotypes [104]. 

We then selected the freeware Beagle to impute genotypes in mixed phased data. Beagle is a Java software that can be redistributed and/or modified under the terms of the GNU General Public License as published by the Free Software Foundation. A copy of the GNU General Public License can be downloaded for academic usage from http://www.gnu.org/licenses/, accessed on 15 March 2022. All the required documentation and the .jar file can be found at https://faculty.washington.edu/browning/beagle/beagle.html#citation, accessed on 15 March 2022. To perform imputation, the genotype file must be converted into variant call format (.vcf) and provided to Beagle with the .jar file. Options to be added with the .vcf file (*gt*) comprehend the reference panel (*ref*), which must have the same genome version as the sample genotype file; the chromosomes involved (*chr*); and the map file (*map*). Information regarding genetic variants and pre-phasing is excluded. Furthermore, the amount of memory available to the Java interpreter can be increased using the *-Xmx* command line argument. If [Mb] is a positive integer, then *-Xmx[Mb]m* sets the maximum amount of memory that will be used by the Java interpreter to [Mb] megabytes. It is helpful to set the *-Xmx* parameter higher than the minimum memory required to analyze your data because having the additional memory available can result in decreased computation time. For customizing other options, e.g., allele frequencies of the estimated markers, we redirect the reader to the official Beagle software guide (released by Brian L. Browning, Department of Medicine Division of Medical Genetics University of Washington and downloadable from the website previously cited in this paragraph).

#### 3.2.3. Imputation with Unphased Genotypes

Alternatively, the imputation can be performed using raw genotypes, i.e., unphased data. Unphased data represent the observed genotypes without regard to which one of the chromosome pairs hold which alleles. With unphased genotypes, HMM parameters are directly estimated using the observed markers [105] and no longer estimated based on reconstructed haplotypes [105]. Data are directly provided to Beagle after the pre-processing QC steps [52], without passing through pre-phasing via Shapeit. This results in a longer computation time because the imputation algorithm must compare allelic frequencies in the study sample to all frequencies in the reference panel to impute the missing genotypes. The pipeline is the same as described in the previous paragraph, with the only difference being that the flag for unphased data in Beagle must be set to *true*.

#### 3.2.4. Quality Control Check after Imputation

QC steps can also be performed after the imputation process, but researchers have to be aware of the fact that QC may discard variants which could provide important insight regarding disease risk. For this reason, filtration strategies on the imputed variants need to be carefully chosen to improve the imputation quality and to reduce the number of variants discarded. Usually, possible checks concerning the presence of Mendelian errors must be performed with related individuals. Other checks may relate to allele and genotype frequencies, excluding monomorphic variants as well as extremely rare imputed variants, depending on the scope of the subsequent analysis. Even if it is very unlikely that samples and markers will be removed based on the call rate during post-imputation filtering, missing rate checks should also be considered. If deviations from HWE in the sample have already been tested in pre-processing steps, as recommended for inferring haplotypes, it is not necessary to perform this check after imputation. On the other hand, as regards the MAF check, according to Charon et al., 2021 [30], the pre-processing step improves the quality of the genotyped SNPs but decreases the number of variants available for imputation by 17.5%. In this study [30], the authors describe a two-step post-imputation filtering process to improve the confidence and the number of very rare and rare imputed variants which considers a less stringent threshold during the pre-processing step.

Eventually, to estimate the imputation accuracy, which mainly increases as the marker density, sample size, or MAF of the reference population increases, some indexes could be calculated. 

There are several key sets of metrics which can be classified into two overarching types: statistics which compare imputed genotypes to ‘gold standard’ genotyped data and statistics produced without reference to true genotypes [88]. Examples of the first type of estimated metrics include the imputation accuracy, concordance rate, squared correlation R^2^, and imputation quality score (IQS) [106,107]. The imputation quality score (IQS) [108] and the Hellinger score [98] can be derived as concordance rate measures. Concordance is defined as the proportion of correctly imputed best-guess genotypes out of all imputed genotypes; it is calculated considering that imputed genotypes can be regarded as “true genotypes”. From this data, a certain proportion of genotypes is then randomly masked and re-imputed. This masking step is repeated several times, hiding arbitrary proportions of genotypes at random, e.g., 200–500 times, and re-imputation is performed on every masked dataset. Eventually the concordance (or the discordance) rate is calculated as the mean of all the concordances (or discordances), estimated with a chosen measure of similarity (or dissimilarity) between the first imputed dataset and the masked and re-imputed ones. The IQS is a concordance rate adjusted for chance with a maximum score of one and no theoretical minimum [109]. An IQS of zero indicates that assigning genotypes randomly according to the true allele frequencies would yield the same proportion of correctly imputed best-guess genotypes. The Hellinger score is a measure of the distance between two probability distributions, the distribution of known genotypes and the distribution of imputed genotypes. It is constructed on a scale of values that ranges between zero and one, with a higher score corresponding to a better imputed genotype. Both measures are useful for assessing imputation quality and can be calculated by setting a random percentage of missing data either for each SNP or in the whole imputed dataset [98].

## 4. Discussion

Imputation from reference panels allows for missing variants from haplotypes of identical or similar sequences to those of genotyped individuals to be imputed [30]. Genotype imputation increases the number of typed variants which increases the statistical power to detect significant associations. 

Despite the presence of a huge variety of scientific research discussing and comparing software implementations for genotype imputation, there is a substantial lack of studies providing a clear and feasible pipeline to be implemented on mixed data, i.e., when the targeted sample comprises both related and unrelated subjects. Considering the difficulty in retrieving extended families, related subjects in mixed data mainly consist of family trios, which were our focus. In this review we have tried to clarify the state of the art [4,110] by reviewing the software and algorithms currently available for imputation in the context of mixed data. We lastly proposed a pipeline that can be applied to both phased haplotypes and on unphased raw genotypes. Specifically, after setting the macro areas for our critical review following the PRISMA guidelines [56] and Grant and Booth guidelines for critical reviews [54], we qualitatively synthesized seven selected works. As we explained, LD-based methods are considered more suitable to estimate haplotypes in presence of closely related subjects, e.g., trios and duos, in our mixed targeted sample context than IBD-based methods. In this research, we do not discuss IBD-based methods due to high percentages of genomic regions shared among closely related individuals.

We considered and recommended the use of the freeware Shapeit for the pre-phasing process, as it can be implemented to accurately estimate haplotypes in both related and unrelated subjects. In an LD-based framework, Shapeit implements HMM by applying Gibbs sampling [47] for inferring the inheritance pattern of each meiosis at all sites across each chromosome and, thus, for reconstructing the haplotypes. Individuals’ haplotypes are updated conditionally using the current estimates of haplotypes from all other samples. Although Shapeit developers recommend imputing non-typed SNPs using the freeware Impute2 [53], which implements an LD-based method, we explored more suitable freeware for mixed type data, since issues may arise when including related subjects in the sample when using Impute2. Suitable freeware included Beagle and fastPHASE, which are both able to work with GWAS-sized datasets. Despite different memory requirements and computational times, the accuracy rates of Beagle and Impute2 are similar [111]. On the other hand, fastPHASE has an even faster computational time than Beagle, but its accuracy is influenced by the proportion of missing genotypes, especially when this proportion is high [112]. Thus, Beagle was finally chosen given its higher accuracy compared to fastPHASE [24,113]. Beagle incorporates a progressive phasing algorithm which is applicable to both to high- and low-frequency variants. For high-frequency variants, it confidently identifies phased heterozygotes at each iteration and fixes the phase of these heterozygotes in subsequent iterations. For data with many low-frequency variants, such as whole-genome sequence data, the method employs a two-stage phasing algorithm that phases high-frequency markers via progressive phasing in the first stage and phases low-frequency markers via genotype imputation in the second stage [52].

Post-imputation QC steps must be followed to assess the accuracy of imputation [24] and control for Mendelian errors. The imputation error rate must be checked considering the familial structured data according to the considerations outlined by Browning and Browning (2022) [102], with detected errors eventually being fixed. In particular, in the presence of related subjects with higher genomic regions in common, incorporating the estimation of haplotypes before the imputation process (pre-phasing step) appears to yield smaller margins of error during genotype reconstruction. Thus, we suggest phasing before performing imputation [114], as the identification of Mendelian errors is easier and the imputation faster than when using raw unphased data. Another important aspect to be considered is the effect of imputation on genomic predictions and their reliability, which is commonly assessed using overall correlations between genomic predictions from observed and imputed genotypes [115]. Controlling for small imputation errors is crucial as they could propagate to the rest of the analysis. Thus, understanding the nature of the data (i.e., related and/or unrelated subjects in the sample) is essential to ensure that the best analysis strategy is implemented. In fact, after genotype imputation, further studies are usually carried out. For example, fine mapping studies [116,117], which can also be combined with constrained regression, e.g., sparse regression [74] and high resolution GWASs, can be used to find causal variants by analyzing the associations between phenotypic traits of interest and genetic variants. Other applications comprise the investigation of regions with loss of heterozygosity or high homozygosity [118,119] in order to understand whether these could represent a risk factor for the investigated disease. 

In this context, the rapid increase of large-scale NGS projects, which has allowed for the deep sequencing of thousands of individuals, means that larger reference panels will soon be made available. This will increase both the number of imputable variants and the number of choices for phasing and imputation software, which will consequently improve the accuracy of imputation [120].

## Figures and Tables

**Table 1:** Qualitative data assessment. In this table, the following data for the initially screened 15 scientific papers are reported: the reference article (col. 1), the type of analysis performed (i.e., phasing, imputation, or both) (col. 2), the type of data that could be processed (col. 3), the software used (col. 4), and the algorithm used (col. 5). The seven papers finally selected to define the pipeline are indicated in bold.

Article	Setting of Scientific Work	Phasing/ Imputation	Data Type	Software	Algorithm
Stephens et al., 2001 [65]	Comparison between algorithms	Phasing	Unrelated/ Extended families/Trios	Algorithms’ description	Expectation Maximization alg./Clarck’s alg./HMM
Browning et al., 2007 [61]	Software and/or algorithm description	Both	Unrelated/ Extended families/ Trios/Duos	Beagle	LD-based Viterbi’s alg. HMM
Delaneau et al., 2008 [58]	Software and/or algorithm description	Phasing	Unrelated/ Extended families/ Trios	Shapeit	LD-based Gibbs’s sampling HMM
Kong et al., 2008 [25]	Comparison between algorithms	Phasing	Unrelated/ Extended families	Algorithms’ description	IBD-based
Yun et al., 2009 [17]	Review of imputation methods	Imputation	Unrelated/ Extended families	Many methods comparison	IBD-based imputation methods
Scheet and Stephens, 2008 [64]	Software documentation	Both	Unrelated/ Extended families/Trios/Duos	FastPHASE	LD-based EM-MC sampling
Hickey et al., 2012 [63]	Software and/or algorithm description	Both	Extended families/ Unrelated	AlphaImpute	IBD-based Long-Range Phasing
Delaneau et al., 2013 [60]	Software and/or algorithm description	Both	Case-control (GWAS) Unrelated	Shapeit1/Shapeit2 Impute2	LD-based Gibbs’s sampling HMM
O’Connell et al., 2014 [55]	Software and/or algorithm description	Both	Unrelated/ Extended Families/Trios	Shapeit	LD-based Gibbs’s sampling HMM
Khankhanian et al., 2015 [62]	Software and/or algorithm description	Imputation	Unrelated	MACH	LD-based HMM
Money et al., 2015 [59]	Software and/or algorithm description	Imputation	Unrelated (GWAS)	LinkImpute	LD-based kNN
Abney and ElSherbiny, 2019 [57]	Software and/or algorithm description	Imputation	Extended families	Kinpute	IBD-based
Delaneau et al., 2019 [15]	Software and/or algorithm description	Phasing	Unrelated/ Extended families/Trios	Shapeit	LD-based Gibbs’s sampling HMM
Shi et al., 2019 [24]	Review of imputation methods	Both	Unrelated/ Extended families/Trios	Many software comparison	Comprehensive assessment of LD-based imputation quality
Browning et al., 2021 [52]	Software documentation	Both	Unrelated/ Extended families/Trios	Beagle	LD-based Gibbs’s sampling HMM
